# A study protocol for an ongoing multi-arm, randomized, double-blind, sham-controlled clinical trial with digital features, using portable transcranial electrical stimulation and internet-based behavioral therapy for major depression disorders: The PSYLECT study

**DOI:** 10.1080/14737175.2022.2083959

**Published:** 2022-06-08

**Authors:** Lucas Borrione, Patricia C Cirillo, Luana VM Aparicio, Beatriz A Cavendish, Leandro Valiengo, Darin O Moura, Juliana P de Souza, Matthias S Luethi, Izio Klein, Bruna Bariani, José Gallucci-Neto, Paulo Suen, Frank Padberg, Stephan Goerigk, Marie-Anne Vanderhasselt, Zhi De Deng, Jacinta O’Shea, Paulo A Lotufo, Isabela M Bensenor, Andre R Brunoni

**Affiliations:** a Service of Interdisciplinary Neuromodulation, Department and Institute of Psychiatry, University of São Paulo Medical School, São Paulo, Brazil; b Laboratory of Neuroscience and National Institute of Biomarkers in Psychiatry, Department and Institute of Psychiatry, University of São Paulo Medical School, São Paulo, Brazil; c Department of Psychiatry and Psychotherapy, University Hospital, LMU Munich, Munich, Germany; d Department of Head and Skin, Faculty of Medicine and Health Sciences, Ghent University, Ghent, Belgium; e Ghent Experimental Psychiatry (GHEP) lab, Ghent University, Ghent, Belgium; f Department of Experimental Clinical and Health Psychology, Psychopathology and Affective Neuroscience Lab, Ghent University, Ghent, Belgium; g Noninvasive Neuromodulation Unit, Experimental Therapeutic & Pathophysiology Branch, National Institute of Mental Health, National Institutes of Health, Bethesda, MD, USA; h Department of Psychiatry and Behavioral Sciences, Duke University School of Medicine, Durham, North Carolina, USA; i Wellcome Centre for Integrative Neuroimaging, Oxford Centre for Human Brain Activity, Department of Psychiatry, Warneford Hospital, University of Oxford, Oxford, UK; j Center for Clinical and Epidemiological Research & Interdisciplinary Center for Applied Neuromodulation, University Hospital, University of São Paulo, São Paulo, Brazil

**Keywords:** Digital mental health, internet-based behavioral therapy, major depressive disorder, portable transcranial direct current stimulation, portable transcranial electrical stimulation

## Abstract

**Background:**

Transcranial electrical stimulation (tES) is considered effective and safe for depression, albeit modestly, and prone to logistical burdens when performed in external facilities. Investigation of portable tES (ptES), and potentiation of ptES with remote psychological interventions have shown positive, but preliminary, results.

**Research design:**

We report the rationale and design of an ongoing multi-arm, randomized, double-blind, sham-controlled clinical trial with digital features, using ptES and internet-based behavioral therapy (iBT) for major depressive disorder (MDD) (NCT04889976).

**Methods:**

We will evaluate the efficacy, safety, tolerability and usability of (1) active ptES + active iBT (‘double-active’), (2) active ptES + sham iBT (‘ptES-only’), and (3) sham ptES + sham iBT (‘double-sham’), in adults with MDD, with a Hamilton Depression Rating Scale – 17 item version (HDRS-17) score ≥ 17 at baseline, during 6 weeks. Antidepressants are allowed in stable doses during the trial.

**Results:**

We primarily co-hypothesize changes in HDRS-17 will be greater in (1) ‘double-active’ compared to ‘ptES-only,’ (2) ‘double-active’ compared to ‘double-sham,’ and (3) ‘ptES-only’ compared to ‘double-sham.’ We aim to enroll 210 patients (70 per arm).

**Conclusions:**

Our results should offer new insights regarding the efficacy and scalability of combined ptES and iBT for MDD, in digital mental health.

## 1 Introduction

Major depressive disorder (MDD) remains a leading cause of disability-adjusted life years (DALYs), despite traditional pharmacological and psychotherapeutic options [1]. MDD still affects more than 300 million people worldwide, with a chronic and recurrent course [2]. First-line treatments for MDD present significant caveats, as antidepressant medications are associated with modest efficacy [3] and adverse effects [4], while in-person cognitive-behavioral therapy (CBT) lacks wide-range availability, and involves higher costs and logistical burdens [5].

Transcranial direct current stimulation (tDCS), the most widely studied format of transcranial electrical stimulation (tES), is a non-invasive brain stimulation technique with moderate effectiveness for the treatment of MDD [6–9]. tDCS delivers continuous and weak electrical currents to the brain, thereby rebalancing neuronal activity and connectivity [10]. According to the neurobiology of MDD, tDCS trials for this condition commonly apply the anode over the left dorsolateral prefrontal cortex (DLPFC) and the cathode over the right DLPFC [6,9,11,12]. The DLPFC is associated with working memory, self-regulation, and decision making, and has been shown to be hypoactive in depression, particularly in the left hemisphere [13]. This leads to a corresponding hyperactivity in the default-mode network, which has been linked with the self-referential and pessimistic ruminations observed in depressive disorders [14,15].

In the past 15 years, several trials showed that tDCS is moderately effective for MDD, suggesting that it could be a first-line intervention, especially in patients with a low-drug resistance [6,9]. However, such an approach is hampered by the limited scalability of tDCS treatment. The relative scarcity of skilled personnel and the logistical burdens and transportation costs associated with daily visits to external facilities are probably associated with its suboptimal utilization in clinical practice. In this context, recent technological advancements are progressively allowing tDCS to be performed remotely, and operated by patients themselves, therefore reducing costs and enhancing scalability. Although this approach sounds appealing, data from home-based trials are still preliminary. A recent open-label pilot study (n = 34) [16] and case series (n = 5) [17] have suggested that home-use tDCS is a feasible intervention for MDD, following necessary precautions and safety guidelines [18]. Furthermore, these studies were open-label and did not present a control arm, limiting the interpretation of their findings.

Concomitantly to these nascent advancements in the practice of home-based tDCS, growing attention has also been directed towards the combination of tDCS and neurobehavioral or psychotherapeutic interventions, aiming to engage the same brain regions of interest, and ultimately, achieve an additive or synergistic therapeutic effect [19,20]. For instance, two pilot, randomized, sham-controlled, clinical trials (RCTs), combining tDCS and cognitive-control training (CCT) for the treatment of MDD, have demonstrated positive, but preliminary results [21,22]. These studies applied a neurobehavioral intervention with tDCS and were performed at research centers.

However, both neurobehavioral and psychotherapeutic interventions can also be delivered remotely, in an internet-based and self-directed manner, especially using interactive smartphone apps [23]. A randomized clinical trial compared an app-based mental health intervention to a clinic-based group intervention, in patients with serious mental disorders, and found higher engagement and acceptability with the app-based intervention, and with similar clinical outcomes [24]. Meta-analyses that evaluated the effect of app-based interventions in MDD found superiority of these interventions over control conditions, with small to large effect sizes [23,25,26], and higher retention rates when there was human feedback and mood assessments through the apps [27].

Moreover, the recent research interest in mental health apps for the treatment of MDD is occurring within a larger framework encompassing the rapid development of digital mental health technologies, in great part, boosted by the social distancing restrictions imposed by the COVID-19 pandemic [28,29]. Therefore, the expansion of digital mental health interventions and their good usability enables better access to healthcare, cost reduction, personalized approaches, and adherence to treatment. While a few studies evaluating the combination of tDCS with CCT have been performed in research facilities, to the best of our knowledge, no controlled trial has investigated the synchronous combination of portable transcranial electrical stimulation (ptES) and a remotely delivered, self-directed and internet-based behavioral intervention (iBT), for the treatment of MDD, in adult patients.

Here, we describe the rationale, study design and methodology of the ongoing *Portable Transcranial Electrical Stimulation and Internet-based Behavioral Therapy for Major Depression Study* (PSYLECT). The main objective of this multi-arm, randomized, double-blind and sham-controlled clinical trial using digital features is to evaluate the efficacy, safety, tolerability, and usability of a combined and synchronous regimen of active ptES and active iBT, as compared with active ptES in monotherapy, and a double-sham regimen, for the treatment of adult patients with MDD. We hypothesize that the combined active treatments will be superior in efficacy, and equally safe and tolerable, when compared to ‘ptES only’ and ‘double-sham.’ The trial started in May 2021 and aims to enroll 210 patients by May 2023.

## 2 Patients and methods

### 2.1 Overview

The PSYLECT trial consists of a randomized, double-blind, sham-controlled clinical trial, in which patients are allocated to one of 3 parallel arms: (1) active ptES + active iBT (‘double active’); (2) active ptES + sham iBT (‘ptES-only’); (3) sham ptES + sham iBT (‘double-sham’). This study was approved by the Ethics Committees of the University Hospital (*Hospital Universitário – HU*) and Clinics Hospital (*Hospital das Clínicas – HC*) of the University of São Paulo, Brazil (CAAE: 13922419.1.0000.0076), according to the principles stated in the Declaration of Helsinki. The trial is registered in clinical-trials.gov (NCT04889976). Before trial entry, all patients provide written and informed consent. The study was designed in March 2019, approved by the Ethics Committees in November 2019, funded in November 2020,, and initiated in May 2021. As of December 2021, approximately 80 patients had already initiated the study and 75 had completed the 6-week treatment period.

In this study, we present 3 co-primary hypotheses: (1) changes in depression scores in the Hamilton Depression Rating Scale 17-item version (HDRS-17) [30], from baseline to endpoint, will be larger in the ‘double active’ compared to the ‘ptES-only’ arm; (2) changes in depression scores (HDRS-17), from baseline to endpoint, will be larger in the ‘double active’ compared to the ‘double-sham’ arm; and (3) changes in depression scores (HDRS-17), from baseline to endpoint, will be larger in the ‘ptES-only’ as compared to the ‘double-sham’ arm. Although fewer co-primary hypotheses could have been theoretically presented, we considered that the 3 of them are sufficiently novel to be appraised, as they (1) test the additive effects of iBT above and beyond ptES, assessing whether the combination is synergistic, (2) evaluate the efficacy of this remotely-performed combination, and (3) enhance the internal validity of the study, considering unexpected biases that could arise in this ‘digital’ trial that might not necessarily be present in on-site trials (as discussed below).

Our secondary hypotheses are that: (1) changes in depression scores will be larger in ‘double-active’ compared to ‘ptES-only,’ in ‘double-active’ compared to ‘double-sham,’ and ‘ptES-only’ compared to ‘double-sham’ arms, using additional depression-rating scales (please refer to these scales below, in item 2.4 Procedures); (2) response (defined as ≥ 50% reduction in HDRS-17) and remission (defined as HDRS-17 ≤ 7) will be larger in ‘double active’ versus ‘ptES-only,’ ‘double-active’ versus ‘double-sham,’ and ‘ptES-only’ versus ‘double sham’ arms; (3) reduction in comorbid anxiety scores will be greater in ‘double-active’ compared ‘ptES-only,’ ‘double-active’ compared to ‘double-sham,’ and ‘ptES-only’ compared to ‘double-sham’; (4) the clinical usability of the ‘double active’ protocol will be regarded as (very) easy by ≥ 80% of recipients, according to a Likert scale specifically created for this original study; (5) all three protocols will be equally safe and tolerable, according to the tDCS Adverse Events Survey, as was observed in two large RCTs involving tDCS for the treatment of MDD, previously completed by this research team [12,31,32].

### 2.2 Patients

We recruit patients of all genders, from ages 18 to 59, with a clinical diagnosis of MDD per DSM-5 criteria (Diagnostic and Statistical Manual of Mental Disorders, 5th edition) [33]. The study is conducted at the University of São Paulo, Brazil, at two of its institutions: the University Hospital (*Hospital Universitário – HU*) and the Institute of Psychiatry of the Clinics Hospital (*Instituto de Psiquiatria – IPq/HC-FMUSP*).

The inclusion criteria are: (1) a current depressive episode of at least moderate severity, with a baseline score on the HDRS-17 [30] ≥ 17; (2) absence of contraindications to tDCS (i.e. metallic plates on the head, brain devices, brain aneurysm clips, cochlear implants, cardiac pacemakers, among others); (3) 8 or more years of formal education; (4) access to a personal smartphone and internet at home; and (5) lifetime refractoriness to no more than 3 antidepressants, at optimal doses and for an appropriate duration of 6 weeks, according to a modified Antidepressant Treatment History Form (ATHF) [34].

The exclusion criteria are: (1) other psychiatric diagnoses (i.e. schizophrenia, schizoaffective disorder, bipolar disorder, obsessive-compulsive disorder, attention-deficit and hyperactivity disorder, eating disorders, personality disorders, substance use disorders), although anxiety disorders, as comorbidities, are accepted; (2) suicidal ideation or a suicide attempt within 4 weeks or less, prior to baseline; (3) previous or current psychotic symptoms, not otherwise specified; (4) depressive symptoms better explained by other clinical conditions (i.e. hypothyroidism, anemia, congestive heart failure, among others) or other psychiatric disorders; (5) severe clinical conditions, including Post-Acute Sequelae of COVID-19 [35]; (6) epilepsy and/or other neurological disorders; (7) suspected or confirmed pregnancy; (8) lactation; and (9) use of diazepam > 10 mg per day (or equivalent doses of other benzodiazepines). Regarding other medications, no antidepressant wash-out is being performed, and antidepressant medications currently in use should be in stable doses for at least 6 weeks prior to baseline.

Patient recruitment is being performed through advertisements in our group website (www.sin.org.br) and in our social media channels (Facebook: neuropsiquiatria.ipq; Instagram: neuropsiquiatria.ipq; LinkedIn: sin-ipq). We also accept clinical referrals from health professionals. Finally, the press offices of the Institute of Psychiatry and of the University of São Paulo Medical School also perform periodic announcements regarding our project, with considerable attention from traditional media.

Our screening process consists of two stages. Firstly, interested volunteers answer an online and confidential survey, available through a secure REDCap (Research Electronic Data Capture) link, in order to provide personal contact information and answer basic multiple-choice questions regarding inclusion/exclusion criteria (Supplementary Material 1), as well as the 9-item Patient Health Questionnaire (PHQ-9) [36]. Subsequently, all survey respondents who have not preliminarily indicated any exclusion criterion, and have achieved a score on the PHQ-9 ≥ 10, are invited for a 45-minute initial online interview, with a research-team psychiatrist, in order to confirm eligibility criteria, according to the Mini-International Neuropsychiatric Interview (MINI) [37]. The two-stage online screening process has proven an asset during the COVID-19 pandemic. Before attending the onsite evaluation, volunteers are screened for COVID-19 symptoms, and if COVID-19 is suspected, a PCR SARS-CoV-2 test is required before the onsite evaluation.

**Figure 1:**
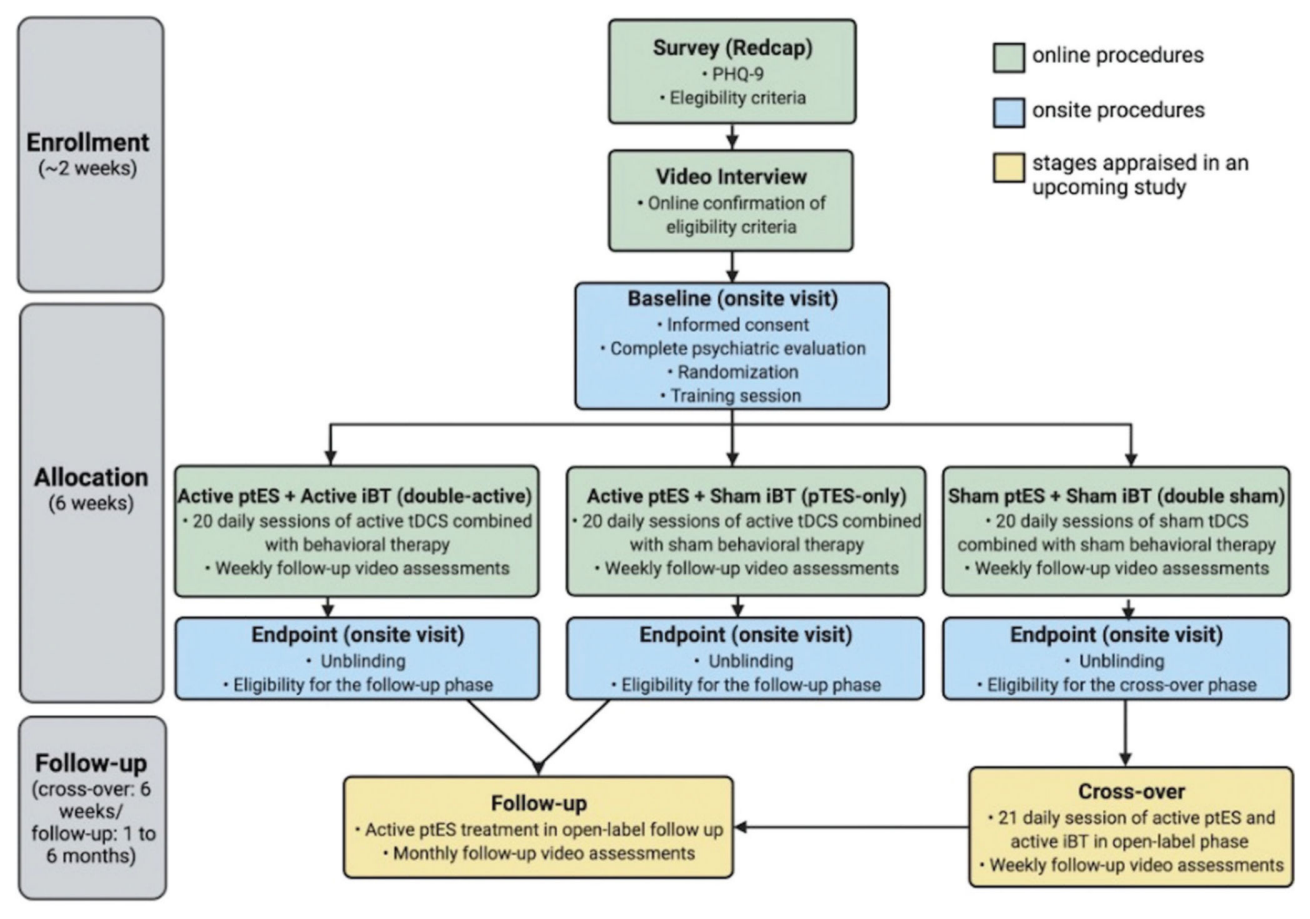
PSYLECT flowchart. Flowchart demonstrating the different and sequential stages of PSYLECT. The initial training session, in the randomized phase, counts as the first session, totaling 21 sessions, according to our study protocol. The cross-over phase is applicable to patients randomized to ‘double-sham’ who do not present response at the endpoint. The follow-up phase is applicable to patients initially randomized to ‘double-active’ and ‘ptES-only’ who present response at the endpoint. (Abbreviations: PHQ-9: Patient health questionnaire-9; ptES: portable transcranial electrical stimulation; iBT: internet-based behavioral therapy; tDCS: transcranial direct current stimulation).

If patients have been considered eligible after these two stages (surveys and online interview), they are invited for an onsite visit in our research centers, for a complete psychiatric evaluation, written and informed consent, baseline assessments, and finally, randomization if enrolled. Moreover, patients will subsequently be invited to receive the two active interventions in: (1) an open-label 6-week crossover phase, if they were previously randomized to ‘double sham’ and do not achieve response at endpoint (defined as ≥ 50% reduction in HDRS-17 scores from baseline to endpoint), and (2) a maximum 6-month open-label follow-up phase if they were previously randomized to ‘double-active’ or ‘ptES-only’ and do achieve response at endpoint. Furthermore, patients who achieve response at the end of the crossover phase are also eligible for the 6-month open-label follow-up phase (Figure 1). A new psychiatric evaluation is performed prior to entering the open-label crossover and follow-up phases, to reconfirm eligibility criteria to undertake active ptES. Finally, patients who were randomized to ‘ptES-only,’ but do not achieve response, will not be eligible for open-label follow-up, as it is unlikely they would profit from a continuation.

### 2.3 Interventions

**Figure 2:**
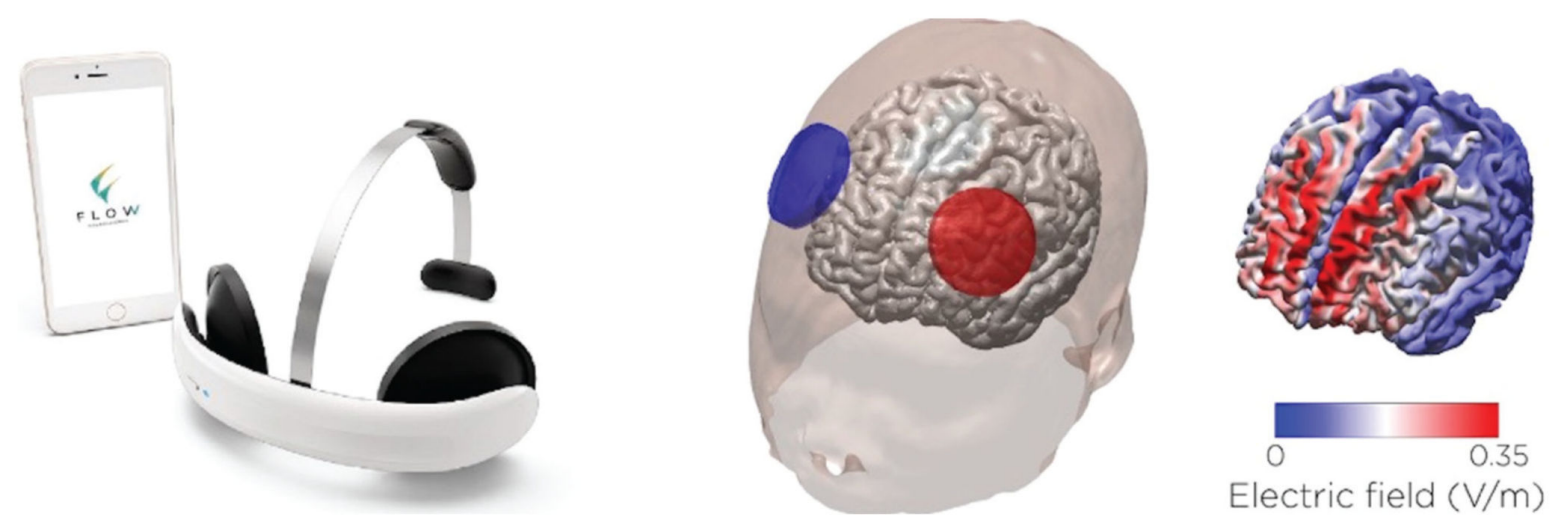
Flow™ device designed for home use. The figure on the left shows the device and supporting iBT app. The figure on the right shows a simulation model of the electrode placement and corresponding electric field distribution on the cortex. The anode and cathode are placed over the left and right prefrontal cortices, respectively. The correct position, in terms of angulation and distance to the eyebrows, is achieved using the app<apos;>s augmented reality feature and the smartphone<apos;>s front camera.

For the combined ptES and iBT, we use tDCS devices and the iBT app developed by Flow Neuroscience™ (Malmö, Sweden) (Figure 2).

The Flow™ ptES device consists of a one-size-fits-all rechargeable headset, with circular electrodes (area = 22.9cm^2^). The anode is placed over the left prefrontal cortex, and the cathode, over the right prefrontal cortex. The device is manufactured with customized, saline pre-humidified and non-reusable sponge pads, which can be easily adjusted over the electrodes, with the aid of circular rubber bands. Flow™ ptES devices are paired, via Bluetooth, to an iBT app (Flow Depression™ – iOS/Android) and provide augmented-reality resources: when patients capture their faces on the smartphone camera, a virtual reality headset is displayed where the actual headset is to be positioned. For PSYLECT, the iBT app has been renamed and the original manufacturer contact information has been replaced with that of our research center.

Flow™ has been approved by health regulatory agencies, for home-use in adult patients with MDD, in the European Union, the United Kingdom, and Brazil (National Agency of Sanitary Health, ANVISA). Moreover, the device has undergone an electric-field (EF) simulation study [38], with 15 brain models obtained from structural MRI from previous clinical trials performed by our team, and displayed compatible EF distributions as compared to those observed in traditional tDCS montages used in those previous studies [31,32]. Furthermore, the Flow device and app have been evaluated in a previous, open-label pilot phase with 5 depressed patients, at our research center, displaying favorable tolerability, safety, and efficacy profiles [17].

For the active tDCS sessions, current strength is delivered at 2 mA (current density = 0.087 mA/cm^2^), for 30 minutes, daily for 5 continuous days (with a subsequent 2-day pause), during the first 3 weeks, and twice-weekly for the following 3 weeks (comprising a total of 21 sessions in 6 weeks). For the sham tDCS sessions, the protocol is similar, but consists of fade-in and fade-out phases of 1 mA, for 45 seconds, at the beginning and at the end of the sessions, with a silent period in between for the remaining 28.5 minutes.

The iBT intervention is performed concomitantly to the tDCS session and consists of an app-based interactive protocol (with a chatbot and online videos), synchronized via Bluetooth, to the tDCS device. For PSYLECT, the chatbot iBT protocol has been translated from English to Portuguese, and all iBT videos are either presented with Portuguese subtitles, or in the case of meditation videos (which allow patients to close their eyes), have been dubbed to Portuguese. Further details about active and sham iBT sessions are not described in this design paper to avoid breaking the blinding of future patients enrolled in this study, with means of access to this publication. The complete details will be provided when the study results are published or upon reasonable request.

### 2.4 Procedures

Upon trial enrollment, patients are initially registered by our research team on the study dashboard, using a personal email as identification. Patients then receive a confirmatory email and are instructed to: (1) download the trial app (in iOS or Android), (2) create their own personal app account and password, which are validated by a trial-provided security code. Randomization (1:1:1) is performed remotely through a computer random generator, using a Mersenne Twister algorithm. Subsequently, patients receive another email for confirmation of trial enrollment, thereby completing the randomization process and trial registration, with preservation of allocation concealment.

Once patients have been enrolled and randomized, their personal accounts become visible for the research team, on the study dashboard, where only the researchers can oversee adherence to, and completion of, home-based trial sessions, all the while remaining blinded. Patients are advised not to describe their app characteristics during the clinical evaluations, for this would unblind the investigators; however, reports of ptES-related adverse events are actively assessed during these online meetings. If unblinding occurs at any point during an evaluation, patients will be referred to another blinded member of the team for the remaining part of the trial. Blinding efficacy assessments for both evaluators and patients are performed at the endpoint.

For the evaluation of depression and anxiety symptoms, we use the following scales: (1) Hamilton Depression Rating Scale, 17-item version (HDRS-17) [30], (2) Montgomery–Åsberg Depression Rating Scale (MADRS) [39], (3) Beck Depression Inventory – II (BDI-II) [40], (4) Positive and Negative Affect Scale (PANAS) [41], (5) State-Trait Anxiety Inventory (STAI) [42], and (6) Hamilton Anxiety Rating Scale (HAM-A) [43]. To evaluate for treatment-emergent manic or hypomanic symptoms and tDCS-related adverse events, the Young Mania Rating Scale (YMRS) [44] and the tDCS adverse events survey [12] are applied, respectively. Our standard tDCS adverse events questionnaire has been previously used and validated in two of our former, completed randomized clinical trials with tDCS in depression [31,32], and covers all common side effects related to tDCS, which are mainly local in nature (i.e. tingling, redness and itching at the application site). Furthermore, the questionnaire also investigates more serious adverse events such as headaches, nausea, and dizziness. All adverse events are graded from mild to severe. Furthermore, at the bottom of this tDCS questionnaire, we provide an open field where participants can freely describe any other adverse events not otherwise captured by the questionnaire, on a weekly basis. We will report all additional adverse events not otherwise captured by the structured tDCS-related adverse events questionnaire, as well as dropout rates in each arm.

Clinical usability of the device is evaluated through a 5-item Likert scale, designed specifically for this trial (which incorporates questions about the ptES device and iBT app) (Supplementary Material 2). The Edinburgh Handedness Inventory – Short Form [45], the Defined Daily Dose (DDD) medication form [46], and sociodemographic and head measurements (head circumference, nasion-inion and tragus-tragus distances) are collected at baseline. The Clinical Global Impression Rating (CGI) form is applied at baseline and endpoint [47] (Table 1).

The first ptES session of each patient is performed at our research centers, with direct supervision of unblinded members of our team, who are not directly involved with clinical evaluations. These members need to be unblinded once they offer consultations regarding app usage. In this initial training session, the different parts of the equipment are presented to the patients, as well as recommendations on when to interrupt the session (i.e. presence of pain or any serious discomfort). If any difficulties are detected during the initial training session, additional onsite training sessions can be scheduled. Once patients are deemed trained for ptES, all further sessions are performed without supervision at the patients’ homes, with recommendations to remain at physical rest (sitting or lying down), throughout the session, in a calm environment and concentrated on the app′s instructions. The app offers a tutorial on how to correctly place the headset, before each individual session, and informs patients to remove the headset after session completion. The app also notifies patients when the headset needs recharging, and sets off reminders for subsequent sessions, which can be performed after a minimum interval of 24 hours. Missed sessions can be rescheduled for the upcoming weeks, with no more than 5 sessions per week.

Patients are evaluated online on a weekly basis by the blinded members of the research staff. Moreover, all trial patients have remote access to supervision upon request by unblinded members of the team. For this purpose, we provide a mobile number for immediate contact. If medical attention is deemed necessary, due to an intercurrence or significant adverse event, an online medical appointment is performed within at most 48 hours. If needed, patients can also be referred to an onsite clinical evaluation at our research centers. Furthermore, adherence and completion of sessions are monitored online, through the study dashboard. Early unblinding and trial discontinuation before study completion are initiated by the following circumstances: (1) significant depression worsening, defined as a > 25% increase in HDRS-17 scores from baseline, during two consecutive weekly assessments; (2) development of suicidal ideation or suicide attempt; (3) development of psychotic symptoms; (4) development of (hypo) mania and/or clinically significant mixed symptoms (YMRS ≥ 8); (5) low adherence to study protocol, defined as < 75% of completed sessions, at any point during the study; (6) absence at any weekly evaluation without justification; and (7) patient consent withdrawal.

After trial completion, the endpoint assessments will be completed by blinded members of our research team. As a final step, patients and clinical evaluators will be unblinded using the study dashboard, and eligibility for the crossover and follow-up stages will be assessed. If participation in the study is terminated at the endpoint assessment, patients are referred to their original treatment facilities.

### 2.5 Digitalization

The Psylect trial has been conceived as a trial with a partial degree of digitization – i.e. although the trial involves onsite evaluations and patient training, it also makes use of online technologies to ‘improve recruitment and retention, data collection, and analytics’ [48]. We perform digital procedures to screen and recruit patients, perform weekly clinical assessments of enrolled patients (from weeks 1 to 5; baseline and endpoint assessments are performed onsite), remotely monitor adherence to the home-based study protocol, receive responses of self-administered surveys and questionnaires (through the REDCap platform). Furthermore, instructional online videos for correct positioning of the headset and Bluetooth connection to the smartphones, for the active and sham iBT interventions, are also offered online.

### 2.6 Data management

Study data are collected and managed using REDCap (Research Electronic Data Capture). REDCap is ‘a secure, web-based software platform designed to support data capture for research studies, providing: 1) an intuitive interface for validated data capture; 2) audit trails for tracking data manipulation and export procedures; 3) automated export procedures for seamless data downloads to common statistical packages; and 4) procedures for data integration and interoperability with external sources [49,50]. REDCap can be installed in a variety of environments, in compliance with international standards, such as HIPAA (Health Insurance Portability and Accountability Act), CFR (Code of Federal Regulations) Title 21/Part 11, FISMA (Federal Information Security Management Act), and Brazilian data management regulations (*Lei Geral de Proteção de Dados – LGPD*).

Data will be coded according to a previously developed data dictionary. Quality of data collection will be monitored by random data quality checks for consistency (e.g. depression scores compatible in different scales) and completeness (absence or few cases of missing data).

### 2.7 Sample size calculation

For Psylect, we estimated a total sample size (n) of 210 randomized patients (70 patients per arm), to achieve the ability to observe statistically significant effects, with a power of 80% (1 − β = 0.8). Dropouts were assumed to increase monotonically (Weibull) and to be equally distributed between arms. We will use 4 measurements (continuous linear change from baseline, weeks 2, 4 and endpoint), between the 3 arms (time × group interaction), in the framework of linear mixed-effects models (LMM) [51,52].

For our sample size calculations, we assumed baseline depression scores and standard deviation (SD) of 25 (±5) on the HDRS-17, distributed equally between groups, based on previous works from our group [31,32,53]. Furthermore, we considered that: (1) placebo effects in the double-sham arm will impact baseline depression scores equal to 1 effect size (ES) in SD units (ES = difference in mean change divided by SD); (2) the ‘ptES-only’ arm will have the same placebo effects present in the double-sham arm (ES = 1), plus a treatment response of ES = 0.4; and, (3) the ‘double-active’ arm will have placebo effects (ES = 1) plus a larger response of ES = 0.8. Therefore, probable endpoint scores of 20, 18 and 16 were used, for ‘double-sham,’ ‘ptES-only’ and ‘double-active,’ respectively. The effect sizes were also chosen based on the results from our previous trials and considering that tDCS and online behavioral therapies have small to moderate effect sizes.

Sample size per arm was calculated based on the smallest detectable group difference (ES tDCS vs. combination = −0.4, ES placebo vs. tDCS = −0.4). Significance levels were Bonferroni corrected for 3-way pairwise comparisons (α = 0.05/3) to control family-wise error rate, while still allowing for more anti-conservative adjustment in the final analysis [54–56].

### 2.8 Statistical analysis

As we have 3 co-primary hypotheses, each co-primary hypothesis will be considered statistically significant if a two-sided p < 0.0167 (Bonferroni-corrected) is obtained. For the analysis of the coprimary outcomes, LLM will be employed, using a first order regressive covariance structure, which includes all observed variables without the need of imputing missing data. The dependent variable is score change on the HDRS-17. Independent variables are time (all observations until week 6), and group (‘double-active,’ ‘ptES-only’ and ‘double-sham’). We will test the statistical significance between the pairwise comparisons per our primary hypotheses. We will employ an intention-to-treat (ITT) approach.

For the analysis of the secondary outcomes, linear mixed-effects models will be employed analogically to the analysis for the primary outcomes. Binary outcomes (response and remission) will be modeled using mixed logistic regression at each timepoint. Improvement in other depressive domains will be evaluated using the same linear hierarchical models described.

## 3 Discussion

PSYLECT will compare the efficacy, safety, tolerability, and usability of: (1) a combined and active regimen of ptES and iBT compared to active ptES in monotherapy; (2) a combined and active regimen of ptES and iBT compared to a double sham counterpart; and (3) active ptES in monotherapy versus a double sham, for the treatment of MDD, in adult patients, for 6 weeks. Antidepressant medications currently in use at baseline will be maintained throughout the trial, in stable doses.

After two RCTs developed by our team that have, respectively, studied the combination of tDCS and an antidepressant in a factorial design [32], and the non-inferiority of tDCS in comparison to an antidepressant [31], in PSYLECT we aim to evaluate the combination of a home-based tDCS protocol with an internet-based behavioral therapy approach.

Neurobehavioral strategies have been increasingly associated with tDCS for the treatment of MDD and other mental disorders, initially inspired by positive results with the combination of tES and motor rehabilitation for the treatment of stroke [19,20]. The neurobiological rationale behind these combinations is based on the ‘state dependency’ of targeted neural circuits [19], with recent evidence suggesting that ongoing neural activity is necessary for tDCS to bring forth neuroplastic effects, especially with concurrent interventions [20]. Previously, Segrave et al. studied the combination of tDCS with cognitive-control training (CCT) in 27 patients with MDD, and observed that the combined treatment demonstrated superior efficacy when compared to either treatment alone, in the reduction of MADRS scores, after 3 weeks [21]. Brunoni et al. (2014) [22] also studied the treatment effect of concurrent tDCS and CCT in 37 patients with MDD. In this study, although both arms (active CCT + active tDCS and active CCT + sham tDCS) displayed similar improvements, it was observed that among patients who received active tDCS, a greater reduction in depressive symptoms was observed in older individuals with better performance in speed and flexibility of information processing, possibly indicating greater engagement of the DLPFC [22]. Recently, another pilot study (n = 31) observed that the combination of tDCS and mindfulness-based cognitive therapy in depressed patients was superior in the maintenance of clinical improvement at a follow-up assessment, as compared to tDCS alone, strengthening the hypothesis that a combined approach could offer therapeutic advantages over tDCS in monotherapy [57].

Despite these advancements in tDCS research and methodology, patient access to the technique is still limited, mainly for logistical reasons. Daily tDCS sessions, performed over several weeks, in clinical or research facilities, can present a challenge to patients, especially in pandemic scenarios [58]. On the other hand, home-use tDCS devices have only recently been developed. Guidelines for conducting research with tDCS at home already exist [18], but few trials have investigated this procedure. An open-label pilot study (n = 34) [16] and a case series (n = 5) [17] have observed that home-use tDCS for MDD could be feasible, safe and tolerable.

In line with the rationale in the paragraph above, we understand that psychological interventions (i.e. neurocognitive interventions, such as psychoeducation or behavioral activation) performed in research facilities could also present similar logistical challenges. In this sense, it is noteworthy that these remote interventions have been used for depression in the last 30 years, according to the available technology in each period (for example, through books, phone calls, software, text messages and, more recently, through smartphone apps). Automated programs are usually carried out through interactive modules and multimedia techniques (photos, advice, sketches, and videos), to engage and motivate patients, generally in an add-on strategy to first-line treatments. Online interventions can be multifaceted, including cognitive training, self-monitoring of mood, mindfulness, psychoeducation, behavioral activation, and cognitive bias modification [59]. A positive trend was observed for fully automated applications and for interventions that provided user feedback (i.e. statistics and progress) [59].

However, one limitation of online psychological interventions [59] is the lack of a clinically verified MDD diagnosis in enrolled patients. This shortcoming was addressed by Josephine et al. (2017), who conducted a systematic review of CBT apps in the treatment of clinically diagnosed MDD [25]. Reviewing data from 19 selected studies, the authors still observed a statistically significant pooled effect size [Hedge<apos;>s g = −0.90, (95% CI: −1.07,−0.73; I2 = 0%)], favoring the active interventions over waiting lists [25]. However, only a small minority of online interventions are supported by controlled studies and many might profit from the ‘digital placebo effect,’ characterized as “placebo-like effects seen from mobile health interventions, such as smartphone apps, and which can influence the overall therapeutic response [60]. In PSYLECT, we use a sham app, instead of no intervention, in consonance with our endeavor to better quantify the digital placebo effect. Furthermore, hundreds of apps are currently available for the treatment of depression, but few studies have been carried out comparing the effect of these internet-based interventions versus sham, in real-life scenarios, and with larger and more heterogeneous clinical samples [61]. Moreover, only a small minority of commercially available mental health apps are supported by evidence from controlled studies [29].

Therefore, the PSYLECT trial builds from accumulated translational knowledge related to the neurobiological rationale of ‘target engagement’ [19], i.e. stimulating the same brain regions of interest with two diverse, but possibly synergistic, interventions: ptES and iBT.

### 3.1 Limitations and challenges

The design and methodology of PSYLECT possesses limitations and challenges worth considering.

Firstly, to the best of our knowledge, this is the first pivotal digital trial involving mobile tES delivered at home. The use of digital features within a trial using remote interventions, such as ours, presents both new opportunities and challenges.

Regarding opportunities, the digital approach can overcome some of the main difficulties of on-site tDCS trials, such as the need of daily visits for several weeks due to treatment schedule and allocating staff for device manipulation. By performing the trial remotely, dislocation burdens are non-existent, as well as the physical need for space at the hospital, and trained staff for delivering tDCS sessions, aspects that increase trial length (usually, the person delivering tDCS sessions can monitor only 2–3 people at once, and physical constraints make delivering several sessions per day difficult). In contrast, a single member of the team can monitor several people using ptES devices at home simultaneously. This aspect is further leveraged by using methods for recruiting potential patients in large catchment areas via social media and Internet.

About challenges, there are many unique, specific methodological aspects that operate differently. Due to the covid-19 pandemic, this trial had to be adapted using digital features to a greater extent than initially planned for, in order not to compromise recruitment and clinical assessment of patients. In this regard, trials with digital features have several distinctive characteristics in terms of randomization, allocation, security and blinding. For instance, randomization and allocation are markedly different in digital trials compared to the traditional SNOSE method (sequentially numbered, opaque, and sealed envelopes) used in many onsite trials. There are also risks of cyber-hacking and data security breaches, making digital clinical trials more vulnerable to these hazards. Moreover, there are concerns that patients can discuss their participation in PSYLECT in online forums (e.g. reddit) or social media, including taking pictures of the app and the device, even if they are instructed not to do so, thereby compromising blinding.

Regarding generalizability, it is unclear whether external validity is increased or decreased in our study. While it could be argued that a greater sample diversity will be achieved due to ease of access to potential patients, it is also possible that a specific population of people are being contacted and enrolled preferentially, representing those more educated, more digitally literate, and more prone to embrace new technologies. It is also unclear whether we should expect higher or lower levels of attrition compared to onsite trials. On the one hand, not needing to return daily to the clinical center could decrease burden and minimize dropouts. On the other hand, patients from digital trials might show less engagement and more difficulties in self-delivering the sessions. The lack of daily contact with the clinical staff might eventually decrease motivation and increase dropouts. All these aspects will be carefully monitored in our ongoing study.

Furthermore, the choice of a single depression rating scale to measure a primary outcome is always challenging, as no single scale covers all psychopathological domains with complete specificity and sensibility [62]. The reasons we have chosen HDRS-17 to measure our primary outcome are the following: (1) this scale is one of the most traditional and widely used scales in clinical trials with depressed participants for the last 50 years, and its scores can be easily adjusted to future meta-analyses involving tDCS for the treatment of MDD; (2) our research team has good experience and inter-rater accuracy using HDRS-17, as it has been previously used as a primary outcome in one large pivotal clinical trial with tDCS for the treatment of MDD[31]; (3) it is a very straightforward and easy to use scale for a clinical trial involving a larger number of participants. Nevertheless, using the MADRS and the BDI-II, to measure other symptom domains (both by clinical raters and participants, respectively), will help us analyze the HDRS-17 outcomes in a wider context.

Finally, this study was designed to evaluate the combined effects of ptES + iBT above ptES, and the effects of ptES above placebo. Although additional study arms (i.e. active iBT + sham ptES) would have expanded the number of hypotheses and comparisons, they would also have increased resources and sample size. Furthermore, the investigation of the effect sizes of tES and iBT separately would best be answered by a factorial design, requiring a completely different approach compared to the present study.

## 4 Conclusions

tDCS has proven a safe, tolerable, and effective strategy for the treatment of MDD. The moderate effect size of the intervention and the logistical issues associated with daily visits to research and/or clinical facilities has created interest in the evaluation of new approaches, such as home-based, remotely supervised regimens and the combination of tDCS with psychological interventions. These new strategies are still in their nascent stages, with preliminary and positive data derived from small, pilot trials. In this regard, PSYLECT will be an RCT with a large sample size, to evaluate the effectiveness, safety, tolerability, and feasibility of ptES associated with iBT, as compared to ptES in monotherapy and double sham, for the treatment of MDD, in adult patients. We understand that the publication of this protocol, before trial completion, provides a positive informative potential to the scientific community, policy makers and funding agencies, thereby enhancing transparency, reproducibility, and accountability of the results.

## Supplementary Material

Supplemental data for this article can be accessed online at https://doi.org/10.1080/14737175.2022.2083959

Supp Info 1

Supp Info 2

## Figures and Tables

**Table 1:** Clinical scales and questionnaires used during PSYLECT.

	Baseline	W1	W2	W3	W4	W5	W6
Sociodemographic data/head measurements	✓						
Edinburgh Handedness Inventory (short version)	✓						
DDD	✓						
HDRS-17	✓		✓	✓	✓		✓
HAM-A	✓			✓			✓
MADRS	✓		✓	✓	✓		✓
CGI	✓			✓			✓
YMRS	✓		✓	✓	✓		✓
Blinding assessment - evaluator							✓
Blinding assessment - patient							✓
PANAS	✓			✓			✓
STAI-T	✓			✓			✓
STAI-S	✓			✓			✓
BDI - II	✓			✓			✓
Likert usability scale		✓	✓	✓	✓	✓	✓
tDCS side effect questionnaire		✓	✓	✓	✓	✓	✓

Abbreviations: DDD: Defined Daily Dose; HDRS-17: Hamilton Depression Rating Scale, 17-item version; HAM-A: Hamilton Anxiety Rating Scale; MADRS: Montgomery-Åsberg Depression Rating Scale; CGI: Clinical Global Impression; YMRS: Young Mania Rating Scale; PANAS: Positive and Negative Affect Rating Scale; STAI-T/STAI: State-Trait Anxiety Inventory; BDI-II: Beck Depression Inventory – II; tDCS: transcranial direct current stimulation.
