# Supplementary material for: A study protocol for an ongoing multi-arm, randomized, double-blind, sham-controlled clinical trial with digital features, using portable transcranial electrical stimulation and internet-based behavioral therapy for major depression disorders: The PSYLECT study
Source: Expert Rev Neurother. Author manuscript; Available in PMC 2026 Jun 2. (PMC10627342; doi:10.1080/14737175.2022.2083959)
Supplement: Supp Info 1 [file EMS212637-supplement-Supp_Info_1.pdf]

Usability Likert Scale_PSYLECT

Dear Volunteer,

Please anwswer the questionnaires found in this link.

Best wishes,

The PSYLECT Team

EQUIPMENT USER EVALUATION

Please, move your cursor to represent to what extent you agree or disagree with each of the assertives on the visual scales below.

1) 1. Placing the transcranial direct current stimulation (tDCS) headset on my head is easy and without complications.

Totally agree I am neutral Totally disagree

(Place a mark on the scale above)

2) 2. Turning on the tDCS device and connecting it to my smartphone is quick and easy.

Totally agree I am neutral Totally disagree

(Place a mark on the scale above)

3) 3. The smartphone app works well, with clear and objective explanations.

Totally agree I am neutral Totally disagree

(Place a mark on the scale above)

4) 4. It is easy to complete the stimulation session within 30 minutes.

Totally agree I am neutral Totally disagree

(Place a mark on the scale above)

5) 5. It is easy to handle and store the equipment until the next session.

Totally agree I am neutral Totally disagree

(Place a mark on the scale above)
