# Supplementary material for: A study protocol for an ongoing multi-arm, randomized, double-blind, sham-controlled clinical trial with digital features, using portable transcranial electrical stimulation and internet-based behavioral therapy for major depression disorders: The PSYLECT study
Source: Expert Rev Neurother. Author manuscript; Available in PMC 2026 Jun 2. (PMC10627342; doi:10.1080/14737175.2022.2083959)
Supplement: Supp Info 2 [file EMS212637-supplement-Supp_Info_2.pdf]

Pre-screening questionnaire (Supplementary Material)

Dear Volunteer,

We are developing a new scientific research with transcranial direct current stimulation (tDCS) for the treatment of depression.

tDCS modulates the function of certain brain areas which might be altered in depression.

tDCS is not painful, does not require invasive procedures and is practically devoid of side effects.

Please, answer the following questionnaire. It is important that you answer these questions yourself, and not a friend, family member and/or caretaker.

We emphasize that this research is only directed to adults between 18 and 59 years of age, with a diagnosis of depression.

Your answers will help us understand if you are elligible to participate in an online medical interview, with one of our researchers of the Institute of Psychiatry of the Medical School of the Clinics Hospital/University of São Paulo.

Address: Rua Dr. Ovídio Pires de Campos, 785, Cerqueira César, CEP 05403-903, em São Paulo-SP.

We further emphasize that these preliminary screening procedures do not guarantee your enrollment in this research, which is based on inclusion criteria and medical evaluation.

The information you share in this questionnaire is protected by medical confidentiality regulations, and will not be shared with persons who do not belong to our research team.

Sincerely yours,

Interdisciplinary Service of Neuromodulation Team

Institute of Psychiatry/Clinics Hospital - Medical School of the University of São Paulo

---

Record ID ______________________________

Date ______________________________

Personal Data

Full Name ______________________________

Gender
- ◯ Female
- ◯ Male
- ◯ Non-binary
- ◯ Neither of the previous alternatives
- ◯ I prefer not to answer

Date of Birth ______________________________

Age ______________________________

Mobile phone number	__________________________________
Personal email	__________________________________<br>(Email you wish to use in order to communicate with our team.)
City of Residency	__________________________________
Brazilian State	◯ AC<br>◯ AL<br>◯ AP<br>◯ AM<br>◯ BA<br>◯ CE<br>◯ ES<br>◯ GO<br>◯ MA<br>◯ MT<br>◯ MS<br>◯ MG<br>◯ PA<br>◯ PB<br>◯ PR<br>◯ PE<br>◯ PI<br>◯ RJ<br>◯ RN<br>◯ RS<br>◯ RO<br>◯ RR<br>◯ SC<br>◯ SP<br>◯ SE<br>◯ TO<br>◯ DF
Do you have a personal smartphone with internet connection at home?	◯ Yes, with connection via cable internet, optic fiber or satellite<br>◯ Yes, but only with my carrier-provided internet (4G)<br>◯ Yes, but only with my carrier-provided internet, and 4G is not available to me<br>◯ No
What is your smartphone's operating system?	◯ iOS (iPhone)<br>◯ Android
If invited, would you be willing to participate in an individual online interview with one of our psychiatrists?	◯ No<br>◯ Yes

When would you be available for this maximum 45-minute online interview? Please check all available time periods.	☐ Tuesdays (10h-12h)<br>☐ Tuesdays (18-20h)<br>☐ Wednesdays (8-10h)<br>☐ Wednesdays (13-17h)<br>☐ Wednesdays (19-20h)<br>☐ Thursdays (8-12h)<br>☐ Thursdays (19-20h)
If invited, would you be willing to come in-person for onsite evalutions, on Mondays and Fridays, from 8-17h, at the Institute of Psychiatry of the Medical School of the University of Sao Paulo (Rua Dr. Ovídio Pires de Campos, 785, Cerqueira Cesar, CEP 05403-903, São Paulo-SP)?	◯ Yes<br>◯ Yes, but only in the mornings<br>◯ Yes, but only in the afternoons<br>◯ No, because I live far away from the Institute of Psychiatry<br>◯ No, because my work schedule does permit<br>◯ No, because I am not comfortable with physical displacements, either because of a medical condition or disability
If our schedule is not compatible with your potential onsite visits, what other time periods and days would you suggest for us?	______________________________
Have you received a diagnosis of depression from any medical doctor?	◯ Yes<br>◯ No
If you have answered "yes" to the previous question, for how long have you had a diagnosis of depression?	◯ 0 to 1 years<br>◯ 1 to 2 years<br>◯ 2 to 5 years<br>◯ 5 to 10 years<br>◯ For more than 10 years<br>◯ I am not sure
Do you take any medications for depression?	◯ Yes<br>◯ No
Would you please list the medications that you take for depression?	______________________________
Have you ever been diagnosed with one of the following conditions?	☐ Bipolarity<br>☐ Bipolar depression<br>☐ Hypomania<br>☐ Mania<br>☐ None of the above
Have you ever received any of the following diagnoses from a medical doctor?	☐ Schizophrenia<br>☐ Alcohol dependence<br>☐ Substance use disorder<br>☐ Personality disorder<br>☐ Hyperactivity and attention-deficit disorder<br>☐ Obsessive-compulsive disorder<br>☐ Others<br>☐ None of the above
Which other diagnoses have you received?	______________________________

Do you have any of the following conditions?	☐ Epilepsy<br>☐ Metal plates in the head<br>☐ Metallic clips in the brain<br>☐ Cochlear implants<br>☐ Cardiac pacemakers<br>☐ Brain tumors or aneurysms<br>☐ Others<br>☐ None of the above
Which other conditions?	__________________________________
Would you like to make any additional observations at this stage?	__________________________________
